# The Effect of Topical Tranexamic Acid on Bleeding Reduction during Functional Endoscopic Sinus Surgery

**Published:** 2017-03

**Authors:** Mohammad Hossein Baradaranfar, Mohammad Hossein Dadgarnia, Hossein Mahmoudi, Nasim Behniafard, Saeid Atighechi, Vahid Zand, Amin Baradaranfar, Sedighe Vaziribozorg

**Affiliations:** 1 *Otorhinolaryngology Research Center, Shahid Sadoughi University of Medical Sciences, Yazd, Iran.*; 2 *Medical Student, Iran University of Medical Sciences, Tehran, Iran.*

**Keywords:** Bleeding, Rhinosinusitis, Polyposis, Tranexamic acid, Topical, Sinus, Surgery

## Abstract

**Introduction:**

Bleeding is a common concern during functional endoscopic sinus surgery (FESS) that can increase the risk of damage to adjacent vital elements by reducing the surgeon’s field of view. This study aimed to explore the efficacy of topical tranexamic acid in reducing intraoperative bleeding.

**Materials and Methods:**

This double-blind, randomized clinical trial was conducted in 60 patients with chronic rhinosinusitis with polyposis (CRSwP) who underwent FESS. Patients were randomly divided into two groups; tranexamic or saline treatment. During surgery, normal saline (400 mL) or tranexamic acid (2 g) in normal saline with a total volume of 400 mL were used in the saline and tranexamic groups, respectively, for irrigation and suctioning. The surgeons’ assessment of field of view during surgery and intraoperative blood loss were recorded.

**Results:**

Mean blood loss was 254.13 mL in the saline group and 235.6 mL in the tranexamic group (P=0.31). No statistically significant differences between the two groups were found in terms of other investigated variables, such as surgical field quality based on Boezzart’s scale (P=0.30), surgeon satisfaction based on a Likert scale (P=0.54), or duration of surgery (P=0.22).

**Conclusion:**

Use of tranexamic acid (2 g in 400 mL normal saline) through washing of the nasal mucosa during FESS did not significantly reduce blood loss or improve the surgical field of view. Further studies with larger sample sizes and higher drug concentrations, and using other methods of administration, such as spraying or applying pledgets soaked in tranexamic acid, are recommended.

## Introduction

Rhinosinusitis, characterized by nasal and sinus mucosal inflammation, is usually associated with symptoms such as facial pain and pressure, nasal congestion, purulent discharge from the nose, hyposmia/anosmia, as well as dental pain and sometimes fever (1). Rhinosinusitis can be divided into acute and chronic subtypes based on duration of symptoms. Chronic rhinosinusitis (CRS) is defined as rhinosinusitis lasting at least 12 consecutive weeks (2). When CRS is resistant to medical therapy, surgery (preferably endoscopic) is indicated (3). Bleeding is one of the most common concerns during endoscopic surgery due to the highly vascular nature of the nasal mucosa and sinuses (4). By reducing the surgeon’s field of view, bleeding increases the risk of damage to adjacent vital elements, prolongs surgery, reduces success rates, and, in some cases, even stops the operation (5). In order to reduce local inflammation and bleeding during surgery, the use of systemic steroids (prednisone) for 5 days in the absence of contraindications is currently recommended prior to surgery (6).

Tranexamic acid is a synthetic derivative of lysine amino acid that inhibits lysine-binding sites on plasminogen molecules, thus blocking fibrinolysis. The anti-fibrinolytic function of tranexamic acid is associated with D-dimer level reduction (7). Tranexamic acid is used for the treatment of primary amenorrhea, bleeding of digestive and urinary systems, thrombo- cytopenia, hemophilia, and Von Willebrand disease, and is also used in cardiothoracic surgery (8). Systemic administration of tranexamic acid can lead to side effects such as blurred vision, dizziness, nausea, vomiting, and headache (9).

However, no evidence has been reported for an increased risk of thrombosis in major surgery (10). In recent years, the topical application of tranexamic acid in cardiac surgery, spinal surgery, total knee arthroplasty, and the treatment of recurrent traumatic hyphema has been the focus of attention (11-14). This study aimed to investigate the effect of topical tranexamic acid on blood loss during FESS in order to advocate its use in such surgeries subject to demonstrable effectiveness and a lack of contraindications. Furthermore, duration of surgery and surgeon assessment of field of view were analyzed.

## Materials and Methods

The current study was conducted after being approved by the Research Ethics Committee of Shahid Sadoughi University of Medical Sciences (ID: 223397, 93/11/4) and obtaining written informed consent from patients. This double-blind, randomized clinical trial was conducted in 2014–2015 in 60 patients suffering from rhinosinusitis with polyposis (CRSwP) who did not respond to medical treatment and who were candidates for endoscopic sinus surgery. The required sample size was estimated to allow a bleeding difference of at least 30 mL to be detected with a confidence interval of 95% and statistical power of 80%.

Patients were randomly divided into two groups (tranexamic group and saline group), each with 30 subjects, using a random number table. Exclusion criteria consisted of previous sinus or nasal surgery, underlying disease with increased risk of thromboses (hypercoagulable states) such as Factor V Leiden, antiphospholipid syndrome, heparin-induced thrombocytopenia, cancer, pregnancy, high blood pressure (systolic >140 mmHg and/or diastolic >90 mmHg), contraindications for the use of tranexamic acid (active clot inside arteries), and patient unwillingness or participation in other similar clinical trials. The degree of sinus involvement in each patient was determined and recorded using the Lund-Mackay scoring system. To reduce inflammation and bleeding, both groups received systemic prednisone 1 mg/kg 5 days before surgery. The anesthesia protocol was the same in both groups (Table.1).

**Table 1:** Anesthesia protocol used in both groups

Premedication	Midazolam 1 mg IV + fentanyl 2.5 micro g/kg IV
**Induction**	Propofol (1.5–2 mg/kg) + atracurium (0.5 mg/kg)
**Maintenance**	Propofol (5–10 mg/kg/h) +atracurium (0.5 mg/kg)every 30 min + N2O %50
**If MAP** ** < ** **80 mm Hg**	TNG drip 0.25-0.5 micro g/kg/min till MAP<80 mm Hg

A throat pack was inserted in each patient in order to prevent blood transferring to the gastrointestinal tract. The surgeon routinely began the procedure after inserting epinephrine 1/2000-soaked pledges into the nasal cavities prior to draping and then injecting 2 mL epinephrine 1/100,000 in the middle turbinate and the anterior junction of the middle turbinate to the lateral nasal wall. During surgery, whenever the field became obscured, irrigation and suctioning were performed. The solution used for this purpose was 400 mL normal saline (saline group) or tranexamic acid 2 g mixed in normal saline with a total volume of 400 mL (tranexamic group). If a greater volume was required, normal saline alone was used. The surgical team were unaware which solution was used for each patient. The dose of tranexamic acid was based on previous studies which investigated the efficacy of 2 g of topical tranexamic acid in total knee arthroplasty and found it effective in reducing blood loss (11,12). During surgery, epinephrine 1/2000-soaked pledges were used when there was excessive bleeding, based on the surgeon preference. Blood-loss volume was calculated by subtracting the volume of irrigation fluid from the total volume collected in the suction container, plus the estimated blood absorbed by the throat pack in each patient. In addition, duration of surgery was recorded for each patient. Based on Boezzart's scale (Table.2), an overall estimate of the surgical field quality was recorded on a scale from 0 to 5 immediately after surgery by the surgeon.

**Table 2:** Surgical Field Quality Based on Boezaart’s scale

Boezaart’s Scale	
**No bleeding**	0
**Minimal bleeding: Not a surgical nuisance and no suction required**	1
**Mild bleeding: Occasional suction required, but does not affect dissection**	2
**Moderate Bleeding: Slightly compromises surgical field, frequent suction required**	3
**Severe Bleeding: Significantly compromises surgical field, frequent suction required, bleeding threat field, just after removal of suction**	4
**Massive bleeding: Prevents dissection.**	5
	

Since the principal aim of the current study was to compare the efficacy of topical tranexamic acid in decreasing intraoperative bleeding and thus improving the surgical field of view, we merely asked the surgeon to report an overall opinion using the Boezzart scale. In addition, surgeon satisfaction in terms of bleeding control and surgical environment were evaluated based on Likert scale from 1 to 5 (very bad, bad, average, good, and excellent) at the end of surgery. During hospitalization, patients were monitored for surgical and drug side effects and the data were recorded. Data were analyzed using SPSS (version 20) using the t-test, Chi-square test, and Mann-Whitney test as appropriate, and a p-value <5% was considered statistically significant.

## Results

The mean age of patients was 40.7 years in the saline group and 38.6 years in the tranexamic acid group (P=0.51). Seventy percent of patients in the saline group and 73.3% in the tranexamic acid group were male (P=0.77). Based on the Lund-Mackay score, the average sinus involvement in the saline and tranexamic acid groups was 16.83 and 15.73, respectively (P=0.355). Mean blood loss was 254.13 mL in the saline group and 235.6 mL in the tranexamic acid group (P=0.314). Based on Boezaart's scale, the mean surgical field quality score was 3 in the saline group and 2.73 in the tranexamic acid group (P=0.305). Based on the Likert scale, the mean surgeon satisfaction score was 3.17 in the saline group and 2.97 in the tranexamic group (P=0.548). Mean duration of surgery was 115.2 and 125.3 minutes in the saline and tranexamic acid groups, respectively (P=0.225).

During surgery and hospitalization, no major complications or noticeable hemodynamic changes were observed in patients, although these factors were under the control of the anesthesiologist during surgery.In general, the results revealed no statistically significant difference between the two groups in terms of the variables investigated (Table.3).

**Table 3:** Results of Patients’ investigated variables

**Variable**	**Group**	**Number**	**Mean**	**P-Value**
**Bloodloss**	Case	30	235.60	0.314
	Control	30	254.13	
**Duration**	Case	30	125.33	0.225
	Control	30	115.17	
**Lund-Mackay**	Case	30	15.73	0.355
	Control	30	16.83	
**Boezzaart**	Case	30	2.73	0.305
	Control	30	3.00	
**Likert**	Case	30	2.97	0.548
	Control	30	3.17	

## Discussion

FESS is indicated for the treatment of CRS that is resistant to medical therapy (3, 13, 14). Since nasal and sinus mucosa are highly vascular, one of the most common concerns during endoscopic surgery is bleeding control (4,15). Tranexamic acid has a known role in the treatment of primary amenorrhea, bleeding of digestive and urinary systems, hemophilia, thrombocytopenia, and Von Willebrand disease (8). Furthermore, it is used topically in cardiac surgery, total knee arthroplasty, spinal surgery, and in the treatment of recurrent traumatic hyphema (16-19). The current study revealed no statistically significant difference between groups regarding the amount of blood loss, surgeon satisfaction, quality of the surgical field of view, and duration of surgery.

Yaniv et al. studied 400 patients who underwent sinus endoscopic surgery in conjunction with septoplasty and conchotomy. Patients who received oral tranexamic acid experienced considerably less bleeding during surgery and afterward. No patients in the tranexamic acid group required additional nasal packs, unlike the saline group in five patients required this intervention (20). The contrast between the Yaniv study and our study may be due to the sample size and method of drug administration (oral intake versus washing). In a study by Jabalameli and Zakeri, 26 out of 56 patients who underwent FESS received topical tranexamic acid (1000 mg in 20 mL of saline), with the remaining patients receiving placebo. Blood loss was less in the tranexamic acid group compared with the placebo group. Given that the size of the groups examined in the Jabalameli and Zakeri study was similar to our own study, the difference between the results may be attributed to the low drug concentration in our study (2000 mg/400 mL saline vs. 1000 mg/20 mL saline used by Jabalameli and Zakeri) (21). In a 2007 study by Athanasiadis, 30 patients undergoing FESS were separated into three groups (2.5 g aminocaproic acid, 100 mg tranexamic acid, or 1 g tranexamic acid sprayed on one side of the nose and saline on the other). No significant bleeding reduction was observed in the aminocaproic acid group. The combined results of both tranexamic acid groups revealed significant improvement in surgical environment at 2, 4, and 6 min after surgery. The application of 1 g tranexamic acid was more effective compared with application of a 100-mg dose (22). The method of tranexamic administration differed in the two studies, with Athanasiadis et al. adopting a spraying technique compared with the washing protocol used in our study. It can be concluded that systemic absorption was greater under the spraying method compared with the washing technique.

In 2009, Moise et al. explored the effect of tranexamic acid on overall blood loss in patients undergoing endoscopic sinus surgery. The total volume of intraoperative and postoperative blood loss was decreased to about half in the group receiving 10 mg/kg tranexamic acid in 10 mL saline solution compared with the group receiving 10 mL of saline solution alone (P=0.0001). Blood loss was three times less after pack removal in the tranexamic acid group (23). The effectiveness of tranexamic acid in the above study and its ineffectiveness in our study may again be due to the method of administration; intravenous versus topical. In 2011, Alimian et al. randomly assigned 84 patients who underwent endoscopic sinus surgeries into two groups. The average blood loss in the group who received 0.1 mL/kg distilled water was significantly greater than that in the tranexamic acid group (P<0.01). Surgeon satisfaction in the tranexamic acid group was also higher than in the distilled water group (P<0.001) (24). These results again confirm the effectiveness of systemic administration of tranexamic acid both in reducing blood loss and in increasing surgeon satisfaction compared with topical administration. 

In another study by Abbasi et al. conducted in 2012 in Iran, 70 patients who were candidates for FESS were randomly placed into two groups of 35. One group received tranexamic acid at a dose of 5 mg/kg and the other group received 15 mg/kg tranexamic acid diluted with saline up to a total volume of 100 mL through intravenous infusion over 10 minutes. The results revealed significant differences between the two groups in terms of surgical field quality and surgeon satisfaction (P<0.05). Moreover, the difference between two groups regarding amount of bleeding was statistically significant (P=0.03) (25). This study also confirms the superiority of systemic administration of tranexamic acid over topical use. In Canada, Langille et al. (2013) evaluated 28 patients with CRS. In the saline group, patients received normal saline intravenously and the intervention group received tranexamic acid diluted in normal saline intravenously. In this study, the rate of bleeding and Wormald grading was recorded. The results showed that adjuvant administration of intravenous tranexamic acid had no significant effect on reducing blood loss and improving surgical field during FESS (26); a conclusion consistent with our own study.

In 2013, Eldaba et al. conducted a study in Egypt in 100 children aged 5–10 years who underwent FESS for CRS. The authors reported that intravenous administration of 25 mg/kg tranexamic acid in 10 mL of saline can decrease blood loss and improve the surgical field. This study also showed that a single dose of tranexamic acid bolus in pediatric patients can improve surgical field quality and decrease amount of blood loss during FESS (27).

In conclusion, the lack of effectiveness of tranexamic acid found in our study in contrast with its effectiveness in most reported studies (with the exception of Langille et al 2013) can be attributed to the method administration, since oral or intravenous administration of tranexamic acid can decrease blood loss and improve surgical field quality during FESS. On the other hand, topical application of tranexamic acid in the form of irrigation during surgery may lead to less systemic absorption and consequently less impact on the amount of bleeding, the surgical field quality, and surgeon satisfaction with the surgical field. Another possible reason for the lack of efficacy in our study may be the frequent resection of cells and left mucosa after each washing which has the effect of creating fresh wound sites with hemorrhagic properties up to the end of surgery.

## Conclusion

Topical use of tranexamic acid (2 g/400 mL normal saline) administered through washing of the nasal mucosa during FESS had no significant effect in terms of reducing blood loss, improving surgical field, or qualitative evaluation of surgeon satisfaction. We would recommend further studies are conducted with larger sample sizes, higher drug concentrations, and other methods of administration such as spraying, or applying tranexamic acid soaked pledges.
